# The computational analyses of handwriting in individuals with psychopathic personality disorder

**DOI:** 10.1371/journal.pone.0225182

**Published:** 2019-12-23

**Authors:** Barbara Gawda

**Affiliations:** Department of Psychology of Emotion & Cognition, Maria Curie Sklodowska University in Lublin, Poland; University of Toronto, CANADA

## Abstract

The main aim of the present study was to examine several parameters of handwriting in order to identify the putative specific patterns of writing associated with psychopathic personality disorder. The hypothesis-generating study was carried out with the use of Mann-Whitney U test to compare two groups of prisoners, without p-value, effect size, and confidence intervals for effect size. The handwriting samples were obtained from two groups of individuals: prisoners diagnosed with psychopathic personality (*n* = 50), prisoners without psychopathic personality disorder (*n* = 30). Two groups were matched in terms of intellectual level, age, and education. The examined handwriting samples were identical. To examine graphical parameters such as structure, proportions, density, inter-spaces, and impulse, the computer programs GlobalGraf were used. This software is employed by Polish Forensic Association. The inter-group comparisons of graphical parameters have shown there is no significant difference (95% confidence intervals for the effect sizes included 0, or negative numbers) in handwriting between prisoners with psychopathic personality disorder and prisoners without this disorder. Logistic regression has been calculated to show whether any handwriting patterns allow to predict psychopathic personality disorder. Results indicate that participants with psychopathic personality disorder do not exhibit significant motor impairments manifesting in structural, density, topographic, proportions, letter spacing, and impulse features of handwriting. This suggests, contrary to many beliefs related to graphology, that psychopathic personality cannot be identified on the basis of computational forensic examination of handwriting.

## Introduction

There is extensive data indicating that motor impairments are associated with mental disorders within schizophrenia spectrum or affective disorders [1–5]. These motor disorders can be manifested in hand movement and measured through handwriting examination tools. However, there is still no conclusive data on the relationship between psychopathy and handwriting. Analyses of the handwriting { XE “handwriting” } of individuals with psychopathy have been of interest to researchers for a long time. Their aim is to establish a profile of handwriting features which would allow identifying a person displaying this disorder. Researchers have been looking for ways to identify psychopathic personality because it is associated with the greatest threat to the respect of law and social rules. However, the research described in the relevant literature on the handwriting of psychopaths shows numerous shortcomings and is not conclusive. Ambiguities regarding the possibility of profiling psychopathy on the basis of handwriting are indicated by Breil [6,7]. In the studies conducted by forensic experts, a number of differences were demonstrated between the graphisms of psychopaths and individuals from control groups. The subjects were selected based on the DSM—IV { XE “DSM- 4” } diagnosis, and in general, over two hundred properties of handwriting were analysed [8]. It has been found that 17 graphical features of handwriting correlated significantly with psychopathic personality traits [8,9]. Despite demonstrated differences, it is not clear whether they are specific to psychopathy because the intellectual level and education were not controlled in these studies; psychopathic persons were prisoners, and the participants of the control group were law students with no criminal record.

Another study with the use of a graphical–comparative method was undertaken to verify the distinctiveness of the graphical features of handwriting { XE “handwriting” } in antisocial people/psychopaths { XE “psychopaths” } [10]. In that study, three groups were compared (in an attempt to deal with the typical shortcomings resulting in non-conclusive data if two groups are taken into account in a study: one comprising prisoners/criminal offenders and second consisting of non-prisoners): two groups of prisoners (one with high antisocial traits and psychopathy, and the second one with a low level of antisocial traits and no psychopathy) and a control group of non-prisoners without psychopathic traits [10]. The studies covered two groups of prisoners meeting the relevant criterion: recidivism, DSM-IV diagnostic criteria such as recklessness, irresponsibility, lack of insight or remorse. The groups were matched in terms of many variables such as intellectual level, education, so as to exclude the influence of factors other than personality pathology traits on graphism. Thus, intellectual level and neurological disorders were controlled; people addicted to alcohol or other intoxicants, as well as those with motor disorders or suffering from other chronic somatic diseases were excluded from examination. The type of text (the same for everyone) and writing conditions (posture while writing, writing instrument, lighting, noise, writing base) were controlled and standardised. Only healthy right-handed people were included in the examination. In this examination of handwriting features, a graphical–comparative method was used. As a result of the analyses it was established that although there are many graphical features differentiating the handwriting of prisoners diagnosed with psychopathy, prisoners without such a diagnosis and the control group, there are few key differences, that is, differences between the two groups of prisoners. The most important element in these findings was comparing the handwriting of both groups of prisoners with psychopathy. In addition, the differences revealed were different from those expected. Prisoners { XE “Prisoners” } diagnosed with psychopathy differ from prisoners without psychopathy in less frequent elements of handwriting such as open oval in the letter ‘a’, a sinusoidal baseline, a cut-off final element of the letter ‘a’, and arcade forms of the letters ‘m’ and ‘n’. The above results were interpreted as correlated with the level of stress experienced by prisoners without a diagnosed psychopathy, and not as specific characteristics of the graphism of psychopathic individuals [10].

Hypothesis. Given the data obtained using the graphical-comparative method, showing some graphical differences between individuals with and without psychopathic personality disorder (PPD), a replication study was undertaken, with the use of a modern computer tool enabling examination of handwriting. This study is not the same as the previous one. This is a replication study involving another sample, taking into account different from the previously examined handwriting parameters, and using the GlobalGraf computer software, not the graphical–comparative method, which is a traditional forensic technique. The computer software was applied to objectivise the measurement of handwriting patterns. This study was undertaken to definitely establish to what extent psychopaths' handwriting contains specific attributes. This study aimed to explain whether the putative differences are specific to PPD and whether it allows to an identification of an individual with psychopathic personality. Due to the fact that psychopathy is linked to serious dysfunctions in experiencing and expressing emotions and that emotional dysfunctions may be associated with motor expression, we may assume that psychopathy may be involved in any handwriting properties [11–13]. Permanent properties of affective difficulties can be manifested in the motor sphere [14]. An example of such association could be psychomotor agitation in a manic state or slowdown in depression [15, 16], or increased muscular tension in psychopaths, i.e. the so-called somatic anxiety with a simultaneous scarcity of mental anxiety [10]. Graphical patterns of handwriting are of motor nature. Thus, a justification for the hypothesis about differences between handwriting of individuals with and without PPD is associated with the presence of increased muscular tension which may impact handwriting movement [13, 14]. Therefore, a research question was formulated: are ther any graphical patterns of handwriting of individuals with PPD which differentiate them from non-PPD individuals. To verify this, the hypothesis-generating study was conducted (Mann-Whitney U test, without p-value, effect size, and confidence intervals for effect size).

## Materials and methods

### Ethics Statement

The participants gave the written consent to participation in the present study according to the guidelines approved by a local Ethics Committee of University of Maria Curie-Sklodowska (no of the protocol 2013/09/PH)

### Participants

Two groups of incarcerated individuals have been examined: the first group—prisoners diagnosed with psychopathic personality disorders (*n* = 50), and the second—prisoners without psychopathic personality disorders (*n* = 30). The participants recruited for the study had been diagnosed by experienced clinicians, and their files contained information regarding the diagnoses as well as their neuropsychiatric disorders, and demographic characteristics. Other data were collected during interviews. Their diagnoses were also confirmed during the examination. The participants were tested using the same diagnostic tools: intellectual level was assessed with the WAIS-R and psychopathic personality disorder with the MMPI-2-RF [17, 18]. All the participants were right-handed. All the participants were criminal offenders (male prisoners), of similar educational background; their mean age was 35.05 years (*SD* = 9.55) (see Table 1).

**Table 1:** Demographic and intellectual characteristics.

Variables	Prisoners with Pd *n* = 50 *M(SD)*	Prisoners without Pd *n* = 30 *M(SD)*	*F*_*(1*, *77)*_
Age	35.54 (6.33)	34.20 (4.97)	.978 ns
Education in years	10.28 (.90)	10.40 (0.85)	.344 ns
Verbal IQ	99.06 (4.84)	97.40 (3.69)	2.60 ns
Digit Span	5.54 (1.21)	5.56 (1.27)	.009 ns
Psychopathic deviate	76.86 (1.16)	38.60 (7.95)	1124.85***

*M*–means, *SD–*standard deviation, ns–non-significant differences

***—significant at the .001 level

With regard to ethnicity, the participants were Caucasian. The subjects were interviewed in five different state prisons. The prisoners, included in the two groups (criterial and control), were similar in terms of conviction for multiple serious crimes against health (offences involving grievous bodily harm), life and public order; felonies, i.e. murder, homicide, aggravated assault, felony assault, assault and battery, assault with weapons, fraud (data collected based on analysis of their files). The population of serious criminal offenders was of interest because of the more frequent occurrence of psychopathic personality disorder among prisoners convicted for multiple crimes compared to other groups of offenders. The purpose was to select groups that were most similar in terms of demographic, intellectual, and neurological characteristics, the only difference being the presence or lack of a psychopathic personality disorder. Thus, inmates with psychotic or neurological impairments were not included in the study. These inmates were not selected because the intention was to examine individuals displaying psychopathic personality disorder without other factors, such as neurological disorders potentially influencing their behaviour.

### Measures

**Fig 1:**
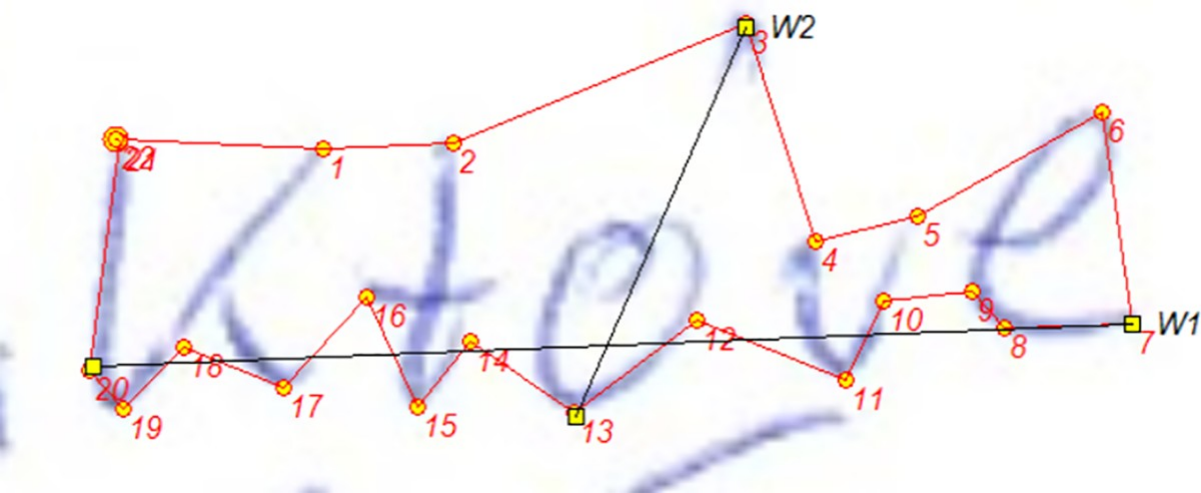
The example of examination with the use of Grafotyp software. Estimation of perimeter (red line between digits 1 and 22), surface of the specimen (the inner part surrounded by a red line), and other parameters such as size proportion (digits denote the order of marking spots, and sections W2 and W1 are necessary to determine the size proportion of the specimen).

**Fig 2:**
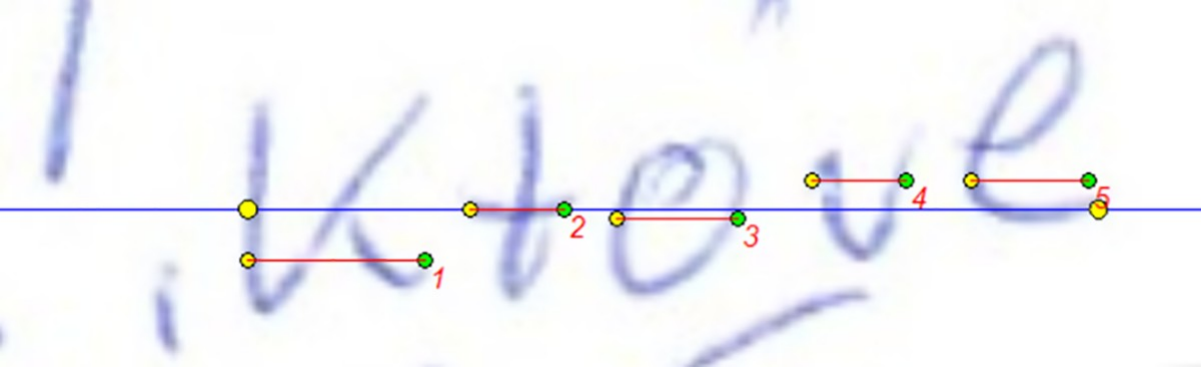
The example of examination with the use of Raygraf software. Estimation of the total width of the specimen (a line between extreme yellow points) and widths of the morphemes of a word (lines no 1, 2, 3, 4, 5).

**Fig 3:**
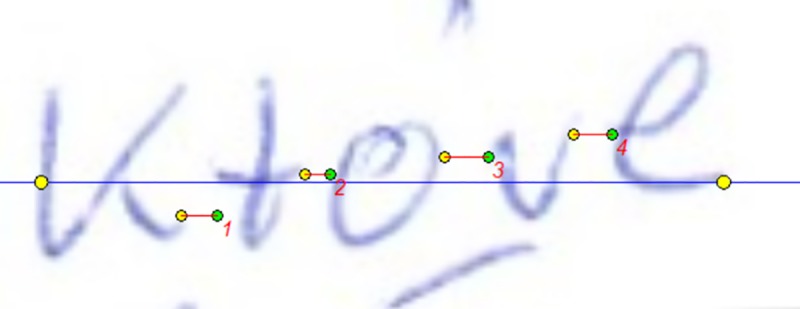
The example of examination with the use of Raygraf software. Estimation of the total width of the specimen (a line between extreme yellow points) and widths of the inter-letter spaces (lines no 1, 2, 3, 4).

- Computational handwriting examination. The examination of the graphical features of handwriting of psychopathic persons with the use of GlobalGraf computer programs was conducted. A Polish team of forensic document examiners has developed a package of computer software called GlobalGraf. The authors of the software are Andrzej Łuszczuk and Krystyn Łuszczuk. The scientific consultants were: Tadeusz Tomaszewski, Mieczysław Goc, Kacper Gradoń. This computer software is distributed by the Polish Forensic Association. It contains four programs: Grafotyp, Raygraf, Kinegraf, Scangraf [19, 20]. The GlobalGraf was used in this study to objectivity of the measurement of the graphical properties of handwriting. Grafotyp is designed for the verification of the structure and size-related parameters of handwriting (an example of the examination with the use of this program is shown in Fig 1).
  Raygraf is a program designed for verification of structural-geometric features of handwriting, in particular the size of line segments, slope, angles, handwriting density and pulse such as the length of selected graphical elements, angles, width of graphical elements, width of spacing between elements (the examples of the examination with the use of this program are shown in Figs 2 and 3), handwriting density and impulse density [21]. Kinegraf is another program which is designed for the verification of kinetic and geometrical parameters of handwriting or signatures. It allows the kinetic-geometrical features of handwriting to be analysed on the basis of graphometric parameters such as the kinetic-geometrical similarity index and the identification value coefficient. Scangraf is a program allowing for the visualisation of the motor features of handwriting by indicating saturation of the surface (paper) with the covering agent. In the Scangraf program, a multiple iteration transformation of bitmaps has been applied to visualise the pressure of the writing instrument against the background, i.e. the „shading” of handwriting [21]. All these programs have been tested for validity. Their accuracy in assessment of the parameters discussed below was confirmed [21, 22].
- The Wechsler Adult Intelligence Scale-Revised (The WAIS-R). A general test of intelligence for adults, based on 11 subtests divided into two parts: verbal and performance. The WAIS-R consists of six verbal and five performance subtests [17]. The verbal IQ and digit span scores were used in statistical comparisons between two groups of prisoners. Reliability of the verbal scale and the digit span subtest in the present study are appropriate; Cronbach’s alpha (verbal IQ) = .897, Cronbach’s alpha (digit span) = .895.
- The Minnesota Multiphasic Personality Inventory (2nd edition) (MMPI-2 = RF). The MMPI-2-RF is a self-report personality assessment tool that contains 567 true/false test items and takes approximately 60 to 90 minutes to complete [18]. The Pd (Psychopathic deviance) scores were used in the statistical analyses to compare the two groups of prisoners and to establish whether any graphical patterns can predict psychopathic deviance. Reliability in the present study: Cronbach’s alpha (Pd) = .899.

### Procedure

Handwriting samples were collected in the standardised condition. All the handwriting samples were collected from men because women diagnosed with psychopathic personality disorder were not included in the study (this disorder is more frequent in men). A total of 80 handwriting { XE “handwriting” } samples were collected, and 960 handwriting assessments were carried out (12 assessments in each specimen). The two groups of participants were matched in terms of demographic parameters, intelligence, as well as presence/lack of visual, motor, and neurological impairments. Control condition for visual, motor, and neurological impairments as a rule is applied in this type of research, and is necessary—given the fact that visual or motor dysfunctions can significantly impact writing movements (see Table 1). The handwriting analysis was carried out for the word ‘które’ (English 'which', see Fig 1). The handwriting samples were earlier subject to identical scaling. All the parameters examined in this study have been described by Goc [21] and the demo of these programs are shown at the website of the Polish Forensic Association. The graphical variables identified in the computer analysis with the use of the GlobalGraf programs comprised the following:

- shape coefficient, which means the “structural parameter of handwriting expressed through the quotient of area of a polygon, constituting the "contour" of the area of the specimen selected for analysis (examined and reference material) by the square circumference of that polygon” [21],
- contour/perimeter of the graphical element/sum of the lengths of sides of the element analysed in the samples, the sum of the length of the sides constituting the outline”contour” of the specimen analysed [21] (see Fig 1),
- surface (area) of the graphical element, the surface area of writing (see Fig 1),
- size proportion, defined as the "quotient of lengths (shorter to longer) of the two most characteristic segments in specimens "A" and "B" (horizontal, vertical or slanting) set according to the same criteria, i.e. these segments should link the corresponding points in specimens "A" and "B”,
- graphotype, i.e., an individual structural feature of handwriting { XE “handwriting” }, that is an individual structural feature of handwriting calculated as a product of the shape coefficient and size proportion structural properties of handwriting, individualising its writer. Mathematically it constitutes the product of the shape coefficient and size proportion [21],
- total width of the specimen analysed, distance between extreme points of the first and last graphical element of the specimen being examined (see Fig 2),
- sum of the widths of the graphemes that make up the element analysed (see Fig 2),
- morpheme density coefficient which is “the quotient of the total width of the sample to the product of the sum of the widths of graphical elements and their number in a given sample” [21],
- letter density coefficient which means the quotient of the morpheme density coefficient to the number of letters/characters in a given specimen,
- sum of inter-letter spaces in the graphical element analysed (see Fig 3),
- number of inter-letter spaces in the graphical element analysed (see Fig 3),
- impulse coefficient denoting the quotient of the total width of the specimen to the product of the sum of the widths of spaces and their number) [21].

### Statistical analysis

First, it was tested whether the handwriting variables were inter-correlated (tau-Kendall coefficient was used as some variables deviated from the normal distribution, see Table 2 and Table 3). The descriptive statistics for these variables are presented in Table 4 and tests of their distributions are shown in Table 5. The reliability of handwriting assessment was examined using test-retest procedure (retest after 4 weeks, tau-Kendall correlations). The key comparisons between the two groups of prisoners were conducted in terms of the grouped variables (U- Mann-Whitney test, SRD as the effect size for Mann-Whitney U test, and confidence intervals for SRD). Finally, logistic regression was calculated to show whether any handwriting parameters allow to predict psychopathic personality disorders.

**Table 2:** Descriptive statistics for the graphical variables identified on GlobalGraf software.

Group	Variables	*M*	*SD*	Min.	Max.
Prisoners with Pd	Shape coefficient	3.33	.72	2.37	5.38
Prisoners without Pd		3.58	.65	2.73	4.93
Prisoners with Pd	Circumference	36.86	6.57	18.79	51.46
Prisoners without Pd		35.34	6.35	26.13	54.70
Prisoners with Pd	Surface	44.79	18.67	11.28	101.31
Prisoners without Pd		44.63	10.60	35.05	70.86
Prisoners with Pd	Size proportion	.46	.12	.27	.87
Prisoners without Pd		.51	.01	.33	.66
Prisoners with Pd	Graphotype	1.47	.50	.79	2.96
Prisoners without Pd		1.83	.69	.87	2.75
Prisoners with Pd	Total width of specimen	12.03	2.76	7.36	20.39
Prisoners without Pd		11.45	1.93	9.41	14.81
Prisoners with Pd	Widths of graphemes	9.15	2.33	5.84	14.23
Prisoners without Pd		9.05	2.13	6.60	12.70
Prisoners with Pd	Mopheme density	.459	.086	.304	.586
Prisoners without Pd	coefficient	.473	.088	.340	.586
Prisoners with Pd	Letter density	.114	.021	.076	.146
Prisoners without Pd	Coefficient	.117	.022	.085	.146
Prisoners with Pd	Sum of inter-letter spaces	2.56	1.21	.76	5.84
Prisoners without Pd		1.90	1.43	.25	4.57
Prisoners with Pd	Number of	2.33	.69	1.00	3.00
Prisoners without Pd	inter-letter spaces	2.16	.71	1.00	3.00
Prisoners with Pd	Impulse	3.39	3.32	.74	14.10
Prisoners without Pd	coefficient	9.96	16.40	1.11	44.88

*M*–mean, *SD*—standard deviation

Groups: prisoners with psychopathic deviate (Pd) and prisoners without this disorder).

**Table 3:** Correlations (τ-Kendall) between handwriting parameters (*n* = 80).

Correlations												
	Shape co	Perimeter	Surface	Size proportion	Grapho type	Total width	Width of graphemes	Morpheme density co.	Letter density co.	Sum of inter spaces	No inter-spaces	Impulse
Shape co	1	-.358**	.058	.133**	.653**	-.311**	-.259**	.155**	.156**	-.193**	-.085	.057
Perimeter	-.358**	1	.879**	-.001	-.192**	.834**	.680**	-.204**	-.119**	.348**	.206**	-.107*
Surface	.058	.879**	1	.048	.077	.780**	.637**	-.140**	-.052	.289**	.166**	-.094*
Size proportions	.133**	-.001	.048	1	.808**	-.267**	-.205**	-.018	.089*	-.126**	.015	.046
Graphotype	.653**	-.192**	.077	.808**	1	-.351**	-.271**	.085	.179**	-.199**	-.049	.076
Total width	-.311**	.834**	.780**	-.267**	-.351**	1	.812**	-.179**	-.123**	.402**	.212**	-.129**
Width of graphemes	-.259**	.680**	.637**	-.205**	-.271**	.812**	1	-.091*	-.071	-.031	-.151**	.149**
Morpheme dens.co	.155**	-.204**	-.140**	-.018	.085	-.179**	-.091*	1	.549**	-.356**	-.478**	.301**
Letter dens. co.	.156**	-.119**	-.052	.089*	.179**	-.123**	-.071	.549**	1	-.196**	-.273**	.143**
Sum of inter-spaces	-.193**	.348**	.289**	-.126**	-.199**	.402**	-.031	-.356**	-.196**	1	.752**	-.644**
No of inter-spaces	-.085	.206**	.166**	.015	-.049	.212**	-.151**	-.478**	-.273**	.752**	1	-.609**
Impulse	.057	-.107*	-.094*	.046	.076	-.129**	.149**	.301**	.143**	-.644**	-.609**	1

**. Correlation is significant at the .01 level (2-tailed).

*. Correlation is significant at the .05 level (2-tailed).

**Table 4:** Descriptive statistics for the grouped-variables.

Variable	Groups	*M*	*SD*	SE
Structure I	Pris.with Pd	102.84	29.73	4.41
	Pris. without Pd	100.51	18.41	3.36
Proportions I	P. with Pd	5.26	1.23	.17
	P. without Pd	5.94	1.39	.43
Density I	P. with PD	.57	.10	.01
	P. without Pd	.59	.12	.03
Inter-spaces I	P. with Pd	4.84	1.74	.25
	P. without Pd	4.07	1.91	.59
Impulse I	P. with Pd	3.39	3.18	.48
	P. without Pd	9.94	14.49	4.73

*M*- mean, *SD–*standard deviation, SE–standard error

**Table 5:** Tests of normality for the variables.

Tests of Normality				
Variable	Kolmogorov-Smirnov^a^		Shapiro-Wilk	
	Statistic	Sig.	Statistic	Sig.
Impulse I	.202	.000	.760	.000
Inter-spaces I	.141	.000	.895	.000
Density I	.178	.000	.787	.000
Proportions I	.062	.000	.974	.000
Structure I	.072	.000	.939	.000

a. Lilliefors Significance Correction

## Results

Table 2 presents correlations between the variables identified with the use of computational assessments (tau-Kendall coefficient). This allows to reduce the number of variables; additionally the inter-correlated variables have been grouped. As it has been shown in Table 3, the variables which correlate highly and significantly were included into the new grouped-variables. Thus, the five grouped-variables have been created. The first one, named *structure*, encompasses surface, perimeter, total width of a specimen, and widths of graphems. The second, named *density*, encompasses morpheme density coefficient and letter density coefficient. The third grouped-variable, named *inter-spaces*, includes number of inter-letter spaces and the sum of inter-letter spaces. The fourth grouped-variable, labelled *proportions*, includes size proportion, graphotype, and shape coefficient. The fifth grouped-variable is named *impulse*. Then, the distributions of these new variables were examined. The descriptive statistics for these variables are presented in Table 4 and data on their distributions in Table 5. As it is shown in Table 5, their distributions deviate from the normal distribution. Thus, non-parametic statistics are used in the inter-group comparisons.

Reliability. The reliability was established by tau-Kendall correlations between the first assessment of handwriting features with the use of the computer software and the second assessment of handwriting features performed with the same computer programs. These correlations are presented in Table 6. They confirmed high reliability of this assessment (τ = .996–1.000) which means that GlobalGraf programs effectively assess handwriting parameters and allow to obtain stable/repeatable, even identical, results.

**Table 6:** Test-retest correlations: Correlations between 1st assessment and 2nd assessment (after four weeks) using the computer software.

Correlations							
	Structure I	Inter-spaces I	Density I		Proportions I		Impulse I
Structure II	.996**	.202**		-.006	-.035	-.050	
Inter-spaces II	.195**	1.000**		-.275	-.136	-.676**	
Density II	-.009	-.275**		.999**	.125	.151*	
Proportions II	-.075	-.136		.125*	.999**	.087	
Impulse II	-.042	-.676**		.151*	.087	.999**	

**. Correlation is significant at the .01 level (2-tailed).

*. Correlation is significant at the .05 level (2-tailed).

To verify whether any significant differences could be identified in the graphical parameters of handwriting between the two groups, i.e. prisoners with PPD and prisoners without PPD, inter-group comparisons were conducted using Mann-Whitney U test, SRD as the effect size, and confidence intervals for SRD. The results of these calculations, presented in Table 7, show there is no significant difference between the two groups in terms of the variables measured by GlobalGraf software. The 95% confidence interval for the SRD (i.e. effect size of Mann-Whitney U test) for impulse parameter included 0, while other 95% confidence intervals for structure, density, inter-spaces, and proportions, included negative numbers. These results mean that there are no significant differences between the two groups of prisoners in their handwriting patterns [23].

**Table 7:** Comparisons between groups of prisoners with Pd (*n* = 50) and without Pd (*n* = 30): U–Mann-Whitney test, SRD effect size.

Variables	U	z	Mean Difference	Std. Error Difference		95% Confidence Intervals of SRD	
					SRD	Lower	Upper
Structure I	276.00	-.222	2.32	9.38	-.632	-.982	-.282
Proportions I	215.00	-1.340	-.67	.40	-.713	-1.413	-.013
Density I	264.00	-.444	-.01	.03	-.648	-.992	-.298
Inter-spaces I	210.00	-1.443	.82	.58	-.720	-1.274	-.166
Impulse I	236.00	-.961	-6.56	2.50	-.685	-1.505	.135

Regression analysis. Finally, a logistic regression was calculated to show whether any handwriting parameters allowed to predict PPD. A logistic regression model, with the enter method based on the likelihood ratio, was applied. The dependent variable was PPD while independent variables included handwriting parameters such as structure, density, proportions, inter-spaces, and impulse. The Hosmer–Lemeshow test was not significant (Σ^2^ = 12.91, *p* = .11) which means that the obtained model is well fitted to the empirical data. The model explains 18.6% of the variance of the dependent variable (Nagelkerke’s pseudo R^2^ = .186). However, the values of partial correlations (ß) for handwriting variables suggest that none of these variables predict PPD (all of them are non-significant, see Table 8). These results definitely confirm there is no handwriting parameter which can be a significant predictor for psychopathic personality disorders.

**Table 8:** Logistic regression model.

Variables in the Equation								
		B	S.E.	Wald (1)	Sig.	Exp(B)	95% C.I. for EXP(B)	
							Lower	Upper
Step 1^a^	Impulse I	.089	.063	1.969	.161	1.093	.965	1.238
	Inter-spaces I	.025	.260	.009	.923	1.025	.616	1.707
	Density I	-1.330	3.307	.162	.687	.264	.000	172.715
	Proportions I	.436	.278	2.464	.117	1.546	.897	2.665
	Structure I	.002	.014	.017	.895	1.002	.975	1.030

a. Variable(s) entered on step 1: Impulse I, Inter-spaces I, Density I, Proportions I, Structure I.

2 Log. Likehood = 52.554

Cox & Snell R^2^ = .111

Nagelkerke R^2^ = .186

## Discussion

**Fig 4:**
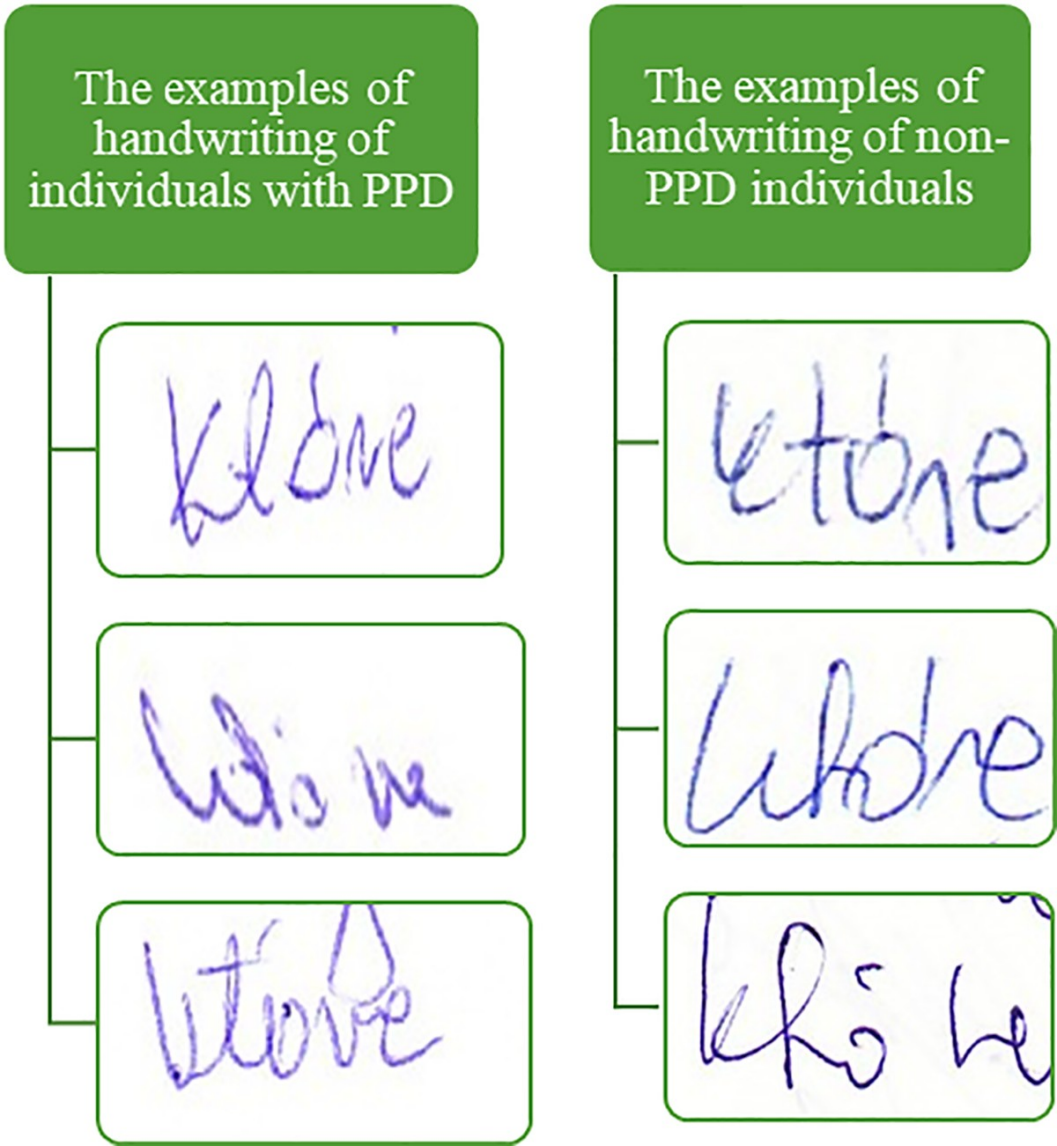
The examples of the analysed handwriting specimens.

The typical comparisons presented in the literature included comparing the characteristics of the handwriting { XE “handwriting” } of a group of prisoners diagnosed with psychopathy and a control group of non-prisoners (men from the general population) without psychopathy. These comparisons reveal usually numerous differences [8, 9]. However, while comparing two groups of prisoners, one with PPD and second without this disorder, no significant difference was found. This means that both psychopathic and non-psychopathic prisoners display similar handwriting patterns such as structure, proportion, density, inter-spaces, and impulse parameters. Differences of key importance in this examination i.e. between prisoners with PPD and without PPD { XE “handwriting” }in handwriting structure, density, topographic, letter spacing, and impulse features were not found. The similarity of their handwriting is illustrated in Fig 4.

The present study confirmed the tendencies already shown in earlier research: no differences found in the compared two groups of prisoners [10]. Most probably, a factor differentiating the handwriting of prisoners and non-prisoners is a synthetic feature, e.g., degree of writing fluency (for example, Goc [21] stresses the importance of synthetic features) or other uncontrolled factors (e.g., micro-damage to the central nervous system) which have a key impact on the differences detected in the handwriting parameters of prisoners and non-prisoners. These differences can be also associated with a prison situation and stress or any negative emotional states influencing the handwriting parameters [24]. The key comparisons between two incarcerated samples showed that no specific parameters measured by GlobalGraf { XE “GlobalGraf” } computer programs were associated with PPD. This means that it is not possible to predict PPD based on the handwriting parameters. These findings are consistent not only with studies related to handwriting in psychopathic individuals but also with research into relationship between other personality traits and handwriting. In contrast to the proponents of personality assessment based on handwriting, previous scientific research did not find associations between handwriting patterns and personality traits [10, 25]. For instance, two studies failed to confirm correlations between the Big Five personality traits and handwriting [26]. Other studies reported results indicating only a minimal value of handwriting analysis in personality assessment [27–30]. The lack of association between handwriting gesture and psychopathic personality disorder can be explained by the fact that motor gesture to a degree is independent from personality [13]. This is emphasized by psychopathologists who argue that a personality disorder does not have to be accompanied by motor/handwriting disorder, and vice versa, a handwriting disorder does not have to be accompanied by a personality disorder. However, there is a possibility that they co-occur.

The lack of relationship between handwriting and PPD can also be viewed from a neuropsychological perspective, where it is emphasised that these two phenomena are very complex in terms of their neurobiological mechanisms and it is difficult to point out clear associations between them [31]. This complex relationship between mental processes and graphomotor features was already presented by A. Luria [32]. Although he did not investigate psychopathic personality disorders, his work published in 1960, entitled *The Nature of Human Conflict*, emphasised various connections between the motor and affective/personality systems. Handwriting, as a manifestation of the motor system, does not reflect emotional or personality traits in the same way in various situations. Luria conducted a number of multiple experiments and reached a conclusion that relationships between motor gesture and emotionality are very complex. For example, motor tension triggered by affect can manifest itself directly as an increase in overall intensity followed by a sudden reduction at the end of the response, or else in a form which is non-specific to changes [32]. It can also be manifested indirectly in a symbolic way or in another form, learned or culturally conditioned. Although handwriting can, to some extent, be modified by emotional states, personality traits, as well as mental or other disorders, this does not mean that personality disorders or other traits are reflected in handwriting in sufficiently diverse ways, to a degree which makes it possible to identify these personality features based on handwriting analysis alone. It seems that these modifications are non-specific to a given disorder.

## Conclusion

To sum up, the data obtained in the study with the use of the GlobalGraf computer software does not indicate the existence of any specific patterns in the handwriting of persons with PPD. This means that the handwriting parameters cannot predict psychopathic personality disorder. However, the findings should be interpreted with cautions due to sample size.

## Limitations

The findings of the present study could be affected by certain limitations. First of all, the sample size could be larger. This, however, might be a difficult undertaking. The current sample of 50 individuals with psychopathic personality disorder is, from a clinical perspective, already rare and relatively large when compared to other studies in the field. Furthermore, it would be of value to compare other samples with psychopathic personality disorders, particularly non-criminal individuals. The optimal solution would be a study taking into account four samples: two groups of incarcerated subjects (with and without PPD), and two groups of non-incarcerated individuals (with and without PPD). Potentially, other comparisons should take into account non-incarcerated subjects, i.e. psychopathic non-prisoners and non-psychopathic non-prisoners. In addition to the varied samples, perhaps it would be of value to use other computational parameters, as well.

## Supporting information

S1 Table This is S1 Table Database. (PDF)

S2 Table This is S2 Table Summary statistics. (PDF)
