# Supplementary material for: The computational analyses of handwriting in individuals with psychopathic personality disorder
Source: PLoS One. 2019 Dec 23;14(12):e0225182. doi: 10.1371/journal.pone.0225182 (PMC7063674; doi:10.1371/journal.pone.0225182)
Supplement: S1 Table — (PDF) [file pone.0225182.s001.pdf]

S1 Table 1. Database

No	Groups	Age	Verbal IQ	Digit Span	Education	Pd	Impulse I	Interspaces I	Density I	Proportions I	Structure I	Impulse II	Proportions II	Interspaces II	Density II	Struture II
1	prisoners with Pd	32	97	6	10	77	2.38	4.78	0.58	5.97	105.41	2.38	5.97	4.78	0.58	102.41
2	prisoners without Pd	32	98	5	11	78	3.21	3.78	0.43	7.84	100.25	3.21	7.84	3.78	0.43	97.56
3	prisoners with Pd	33	96	6	11	79	2.09	4.8	0.59	4.21	94.15	2.09	4.21	4.8	0.59	94.15
4	prisoners with Pd	34	96	4	9	78	1.72	5.56	0.53	6.13	99.64	1.72	6.13	5.56	0.53	97.64
5	prisoners with Pd	35	95	5	9	75	0.74	6.31	0.73	5.41	43.78	0.74	5.41	6.31	0.73	46.78
6	prisoners without Pd	36	94	6	11	40	1.11	7.57	0.49	4.15	144	1.11	4.15	7.57	0.49	144.76
7	prisoners without Pd	35	100	5	11	41	44.88	1.55	0.38	5.5	98.06	43.58	5.2	1.55	0.38	98.06
8	prisoners with Pd	37	101	4	9	79	1.53	6.56	0.5	5.19	120.19	1.53	5.19	6.56	0.5	120.19
9	prisoners without Pd	34	100	4	11	38	1.49	5.28	0.33	4.18	93.71	1.49	4.18	5.28	0.32	93.71
10	prisoners with Pd	35	102	5	11	76	14.1	4.76	0.38	4.29	83.93	13.4	4.09	4.76	0.38	83.93
11	prisoners with Pd	29	101	7	10	78	3.39	4.03	0.61	4.92	116.2	3.39	4.92	4.03	0.61	116.1
12	prisoners with Pd	25	104	5	10	76	3.57	3.53	0.52	4.93	97.66	3.57	4.93	3.53	0.52	98
13	prisoners with Pd	24	105	5	10	79	3.7	4.54	0.51	4.79	139.45	3.7	4.79	4.54	0.51	139.45
14	prisoners with Pd	26	103	5	11	76	1.13	8.84	0.46	4.6	193.87	1.13	4.6	8.84	0.46	190.03
15	prisoners with Pd	27	98	6	11	76	11.07	2.02	0.71	6.24	123.27	11.02	6.24	2.02	0.71	123.27
16	prisoners with Pd	48	103	5	11	78	1.09	6.81	0.69	6.73	97.05	1.09	6.73	6.81	0.68	90
17	prisoners with Pd	46	102	7	10	75	7.69	2.52	0.6	6.23	95.48	7.69	6.23	2.52	0.6	95.48
18	prisoners with Pd	35	103	5	10	75	0.89	7.57	0.73	5.08	95.3	0.89	5.08	7.57	0.73	97.01
19	prisoners with Pd	58	113	6	11	76	1.03	6.3	0.41	6.48	72.9	1.03	6.18	6.3	0.4	72.9
20	prisoners with Pd	36	102	7	9	78	1	6.05	0.73	8.89	83.41	1	8.89	6.05	0.73	89.01
21	prisoners without Pd	37	101	5	11	38	3.37	2.76	0.43	7.58	97.74	3.37	7.58	2.76	0.43	96.74
22	prisoners with Pd	35	103	6	11	79	1.86	5.28	0.44	5.46	92.53	1.86	5.46	5.28	0.44	92.53
23	prisoners with Pd	34	98	5	10	76	2	4.78	0.6	3.66	128.27	2	3.66	4.78	0.6	128
24	prisoners with Pd	40	96	5	11	78	1.24	6.56	0.69	4.3	119.68	1.24	4.3	6.56	0.67	119.68
25	prisoners with Pd	39	112	6	10	76	2.05	4.29	0.51	4.14	81.31	2.01	4.14	4.29	0.51	81.31
26	prisoners with Pd	39	100	6	11	77	2.77	4.29	0.52	5.7	108.73	2.77	5.7	4.29	0.52	100.7
27	prisoners with Pd	37	99	7	9	77	6.88	2.76	0.67	3.88	69.98	6.1	3.88	2.76	0.67	69.98

28	prisoners with Pd	32	93	6	11	78	3.5	4.03	0.66	6.9	128.02	3.5	6.9	4.03	0.66	126.02
29	prisoners with Pd	31	97	5	11	77	4.1	3.52	0.44	4.45	101.52	4.1	4.45	3.52	0.44	101.52
30	prisoners without Pd	30	98	7	12	35	2.71	3.78	0.59	4.93	109.14	2.71	4.93	3.78	0.58	109.14
31	prisoners with Pd	37	96	6	10	79	2.38	4.78	0.58	6.79	106.41	2.38	6.79	4.78	0.58	106.41
32	prisoners without Pd	32	96	5	11	36	5.21	3.78	0.43	7.84	100.25	5.11	7.84	3.78	0.43	100.25
33	prisoners with Pd	34	95	4	8	76	2.09	4.8	0.59	5.79	86.05	2.09	5.79	4.8	0.57	86.05
34	prisoners with Pd	34	94	6	11	76	1.72	5.56	0.53	5.65	57.84	1.72	5.65	5.56	0.53	55.84
35	prisoners with Pd	27	92	5	10	76	0.74	6.31	0.73	5.41	43.78	0.74	5.41	6.31	0.73	43.78
36	prisoners without Pd	46	101	5	11	42	11.11	7.57	0.49	6.15	144.76	11.11	6.15	7.57	0.49	142.76
37	prisoners without Pd	24	100	6	10	42	44.88	1.25	0.69	6.48	98.06	40.78	6.48	1.25	0.69	98.06
38	prisoners with Pd	25	102	5	11	78	1.53	6.56	0.5	5.06	130.19	1.53	5.06	6.56	0.5	130.19
39	prisoners without Pd	25	101	5	10	40	1.49	5.28	0.62	6.19	82.59	1.49	6.19	5.28	0.62	82.59
40	prisoners with Pd	41	94	6	11	76	14.1	1.76	0.38	4.92	80.25	14.1	4.92	1.76	0.38	80.25
41	prisoners with Pd	43	95	5	10	75	3.39	4.03	0.61	4.37	107.88	3.39	4.37	4.03	0.61	107.12
42	prisoners with Pd	46	98	4	10	76	3.57	3.53	0.52	4.93	97.66	3.57	4.93	3.53	0.52	99.66
43	prisoners with Pd	40	98	1	11	77	3.7	4.54	0.51	4.03	148.32	3.4	4.03	4.54	0.51	144.02
44	prisoners with Pd	39	90	6	11	76	1.13	8.84	0.46	4.15	193.87	1.13	4.15	8.84	0.46	191
45	prisoners without Pd	26	93	1	11	39	16.07	2.02	0.71	6.2	123.27	15.17	6.2	2.02	0.71	119.01
46	prisoners without Pd	40	97	8	10	38	1.09	6.81	0.69	6.6	97.05	1.09	6.6	6.81	0.69	96.01
47	prisoners with Pd	39	98	4	10	77	7.69	2.52	0.6	5.27	88.43	7.69	5.27	2.52	0.6	88.43
48	prisoners with Pd	39	96	5	11	76	0.89	7.57	0.73	5.08	81.4	0.89	5.08	7.57	0.73	81.4
49	prisoners without Pd	30	96	5	10	39	1.03	6.3	0.41	6.14	67.74	1.03	6.14	6.3	0.41	67.74
50	prisoners with Pd	37	95	6	11	77	1	6.05	0.73	8.89	83.41	1	8.89	6.05	0.73	83.41
51	prisoners without Pd	31	94	6	9	40	16.37	2.76	0.73	7.58	87.74	16.37	7.58	2.76	0.73	92.74
52	prisoners with Pd	34	100	7	11	78	1.86	5.28	0.44	4.46	92.53	1.86	4.46	5.28	0.44	92.53
53	prisoners with Pd	37	95	6	8	76	2	4.78	0.6	3.66	128.27	2	3.66	4.78	0.6	128.27
54	prisoners with Pd	35	96	6	8	76	1.24	6.56	0.69	4.3	119.68	1.24	4.3	6.56	0.69	119.68
55	prisoners with Pd	29	103	7	11	76	2.05	4.29	0.51	4.14	81.31	2.05	4.14	4.29	0.51	81.31
56	prisoners with Pd	40	97	6	11	77	2.77	4.29	0.52	5.7	108.73	2.77	5.7	4.29	0.52	108.73
57	prisoners with Pd	35	104	5	11	78	2.88	2.76	0.67	3.88	69.98	2.88	3.88	2.76	0.67	69.98

58	prisoners with Pd	39	100	8	11	76	3.5	4.03	0.66	6.9	128.02	3.5	6.9	4.03	0.66	128.02
59	prisoners with Pd	33	95	6	10	76	4.1	3.52	0.44	4.24	101.52	4.1	4.24	3.52	0.44	101.52
60	prisoners without Pd	27	95	7	10	40	2.71	3.78	0.59	4.93	89.15	2.71	4.93	3.78	0.59	87.99
61	prisoners with Pd	29	95	4	10	77	4.1	3.52	0.44	4.24	101.52	4.1	4.24	3.52	0.44	101.52
62	prisoners with Pd	28	94	8	11	76	2.05	4.29	0.51	4.14	81.31	2.05	4.14	4.29	0.51	86.39
63	prisoners with Pd	39	100	4	11	78	6.37	2.76	0.73	7.58	87.74	6.37	7.58	2.76	0.73	90.74
64	prisoners with Pd	36	101	7	11	77	1.11	7.57	0.49	4.15	144.76	1.11	4.15	7.57	0.49	144.76
65	prisoners with Pd	35	111	6	9	78	9.88	1.25	0.69	6.48	98.06	9.88	6.48	1.25	0.69	98.06
66	prisoners without Pd	35	102	5	10	39	7.11	7.57	0.49	4.15	144.76	7.11	4.15	7.57	0.49	156.76
67	prisoners without Pd	36	101	7	11	38	44.88	1.25	0.69	6.48	98.06	44.88	6.48	1.25	0.69	98.06
68	prisoners without Pd	29	104	6	11	36	1.49	5.28	0.62	4.18	83.71	1.49	4.18	5.28	0.62	83.71
69	prisoners without Pd	34	105	6	11	36	6.37	2.76	0.73	7.58	87.74	6.37	7.58	2.76	0.73	87.74
70	prisoners without Pd	40	103	7	11	37	1.49	5.28	0.62	4.18	101.34	1.49	4.18	5.28	0.62	101.34
71	prisoners without Pd	39	95	5	10	36	6.37	2.76	0.73	7.58	87.74	6.37	7.58	2.76	0.73	87.74
72	prisoners without Pd	39	94	6	11	35	2.71	3.78	0.59	4.93	89.15	2.71	4.93	3.78	0.59	89.15
73	prisoners without Pd	37	95	7	10	37	44.88	1.25	0.69	6.48	98.06	44.88	6.48	1.25	0.69	98.06
74	prisoners without Pd	37	93	5	11	36	1.49	5.28	0.62	4.18	102.71	1.49	4.18	5.28	0.62	102.71
75	prisoners without Pd	35	94	6	10	38	6.37	2.76	0.73	7.58	87.74	6.37	7.58	2.76	0.73	87.74
76	prisoners without Pd	34	96	7	11	34	1.49	5.28	0.62	4.18	103.71	1.49	4.18	5.28	0.62	103.71
77	prisoners without Pd	37	91	5	9	30	6.37	2.76	0.73	7.58	87.74	6.37	7.58	2.76	0.73	87.74
78	prisoners without Pd	35	95	5	10	32	2.71	3.78	0.59	4.93	89.15	2.71	4.93	3.78	0.59	86.99
79	prisoners without Pd	34	96	5	9	34	1.49	5.28	0.62	4.18	99.74	1.49	4.18	5.28	0.62	99.74
80	prisoners without Pd	40	94	5	8	34	6.37	2.76	0.73	7.58	119.94	6.37	7.58	2.76	0.73	115.9
