# Supplementary material for: The computational analyses of handwriting in individuals with psychopathic personality disorder
Source: PLoS One. 2019 Dec 23;14(12):e0225182. doi: 10.1371/journal.pone.0225182 (PMC7063674; doi:10.1371/journal.pone.0225182)
Supplement: S2 Table — (PDF) [file pone.0225182.s002.pdf]

S 2 Table 2. Summary statistics

Summary statistics

		age	Verbal IQ	Digit span	Education in years	Psychopathy deviate	Impulse I	Inter-spaces I	Density I	Proportions I	Structure I
N	Valid	80	80	80	80	80	80	80	80	80	80
Mean		35.03	98.43	5.55	10.32	62.51	5.84	4.55	.58	5.51	101.94
Std. Error of Mean		.65	.50	.13	.09	2.15	1.08	.20	.01	.14	2.90
Median		35.00	98.00	6.00	11.00	76.00	2.71	4.41	.59	5.13	98.06
Std. Deviation		5.86	4.49	1.23	.88	19.27	9.67	1.83	.11	1.33	25.96
Variance		34.41	20.22	1.51	.77	371.51	93.58	3.36	.01	1.77	674.22
Minimum		24.00	90.00	1.00	8.00	30.00	.74	1.25	.33	3.66	43.78
Maximum		58.00	113.00	8.00	12.00	79.00	44.88	8.84	.73	8.89	193.87

Summary Statistics continued

		Impulse II	Proportions II	Inter-spaces II	Density II	Structure II
N	Valid	80	80	80	80	80
Mean		5.74	5.50	4.55	.57	101.74
Std. Error of Mean		1.04	.14	.20	.01	2.86
Median		2.71	5.13	4.41	.59	98.03
Std. Deviation		9.38	1.33	1.83	.11	25.60
Variance		88.11	1.77	3.36	.01	655.37
Minimum		.74	3.66	1.25	.32	43.78
Maximum		44.88	8.89	8.84	.73	191.00
